# Urinary Metabolic Biomarker and Pathway Study of Hepatitis B Virus Infected Patients Based on UPLC-MS System

**DOI:** 10.1371/journal.pone.0064381

**Published:** 2013-05-16

**Authors:** Aihua Zhang, Hui Sun, Ying Han, Guangli Yan, Xijun Wang

**Affiliations:** National TCM Key Lab of Serum Pharmacochemistry, Key Lab of Chinmedomics, Heilongjiang University of Chinese Medicine, and Key Pharmacometabolomics Platform of Chinese Medicines, Harbin, China; Drexel University College of Medicine, United States of America

## Abstract

Hepatitis B virus (HBV) is the fatal consequence of chronic hepatitis, and lack of biomarkers has been a long standing bottleneck in the clinical diagnosis. Metabolomics concerns with comprehensive analysis of small molecules and provides a powerful approach to discover biomarkers in biological systems. Here, we present metabolomics analysis applying ultra-performance liquid chromatography/electrospray ionization quadruple time-of-flight mass spectrometry. (UPLC-Q-TOF-HDMS) to determine metabolite alterations in HBV patients. Most important permutations are elaborated using multivariate statistical analysis and network analysis that was used to select the metabolites for the noninvasive diagnosis of HBV. In this study, the total 11 urinary differential metabolites were identified and contributed to HBV progress involving several key metabolic pathways by using pathway analysis with MetPA, which are promising biomarker candidates for diagnostic research. More importantly, of 11 altered metabolites, 4 metabolite markers were effective for the diagnosis of human HBV, achieved a satisfactory accuracy, sensitivity and specificity, respectively. It demonstrates that metabolomics has the potential as a non-invasive tool to evaluate the potential of these metabolites in the early diagnosis of HBV patients. These findings may be promising to yield a valuable insight into the pathophysiology of HBV and to advance the approaches of diagnosis, treatment, and prevention.

## Introduction

Hepatitis B continues to be a worldwide clinical problem with approximately 360 million people chronically infected [1]. In China, about 120 million people are carriers of HBV, accounts for 30% of hepatic cirrhosis globally [2]. During a five-year period, 10%–20% of patients with chronic hepatitis develop cirrhosis [3]. Cirrhosis precedes most cases of hepatocellular carcinoma (HCC), with 70%–90% of HCC developing from a background of chronic liver cirrhosis [4]. These data clearly indicate the critical importance of early diagnosis of hepatic cirrhosis. Although liver biopsy (LB) is currently recommended as the gold standard method of staging fibrosis in patients with chronic HBV, it has several disadvantages such as poor patient compliance, sampling error, limited usefulness for dynamic surveillance and follow-up. Therefore, a reliable, noninvasive diagnostic system to predict and assess treatment and prognosis of liver cirrhosis is needed. Among them, metabolomics analysis is a powerful tool to advance the diagnosis, treatment, and prevention of human diseases [5], [6].

The field of metabolomics continues to grow rapidly over the last decade and has been proven to be a powerful technology in predicting and explaining complex phenotypes in diverse biological systems, might be the sole technology capable of detecting complex, biologically essential changes [7]. Metabolomics involves the establishment of relationships between phenotype and a metabolic signature, which are key aspects of biological function. Recent developments have suggested that understanding changes in metabolite profiles will confer a high degree of predictive accuracy in terms of understanding the fundamental mechanisms resulting in perturbations of the metabolic state [8]–[11]. As such, metabolomics is fast becoming an important discovery tool for new diagnostic and prognostic biomarkers. It is envisioned that this will provide new avenues towards preventive medicine and prognostic strategies for tailored therapeutic and personalized nutrition management. The development of metabolomics approaches and the new generation of biomarker patterns will provide the opportunity to associate complex metabolic regulations to disease [12]–[15]. It is hoped that the information derived from metabolite profiling will make it possible to suggest individualized therapies that more effectively treat disease. Metabolomics has been used to characterize metabolic signatures for various diseases including depression, cancers, diabetes, Parkinson's disease and Alzheimer's Disease [16].

Traditional markers of classical clinical chemistry and histopathology method are not region-specific and only increase significantly after substantial disease injury [17]–[19]. Therefore, more early markers of disease are eagerly needed. Incidence of HBV is rising at a rapid rate and the sensitivity and specificity of the clinical diagnosis of HBV is quite low [20]. However, sensitivity of current diagnostic markers is relatively low, difficult to get outcome immediately and not particularly effective in separating cases of HBV from other non-HBV disorders. Fortunately, the rapid development of metabolomics technology platforms has been used to explore the particular metabolites, potentially diagnostic and prognostic biomarkers for deep understanding the essence of HBV. This paper was designed to investigate a comprehensive metabolome of patients HBV-induced cirrhosis by UPLC-Q-TOF-HDMS combined with pattern recognition methods to identify urine biomarkers for HBV, explore the diagnostic possibilities, and enhance the understanding of its mechanisms.

## Materials and Methods

### Ethical Statement

Signed and informed consent was obtained from each participant, and the project was approved by the ethics committee of Heilongjiang University of Chinese Medicine, and was conducted according to the Declaration of Helsinki Principles.

### Subjects

Patients were collected from Hospital of Heilongjiang University of Chinese Medicine, China. HBV (n = 13) patients and healthy volunteers (n = 11) were recruited in this study. The outcomes of Health Survey Questionnaire in patients with HBV and the normal controls were assessed, and the related clinical information including gender, age, body mass index, basic syndromes of disease and main parameters of liver makers were collected in Table S1. Exclusion criteria are that patients (nonsmoker) had cancer, cardiac insufficiency, renal inadequacy, respiratory failure, alimentary tract hemorrhage, or other diseases that will affect the clinical observations and biological indicators. These HBV patients had not took any medicines or supplements before they collected urine samples.

### Chemicals and reagents

Acetonitrile, HPLC grade, was obtained from Merck (Darmstadt, Germany); methanol (HPLC grade) was purchased from Fisher Scientific Corporation (Loughborough, UK); water was produced by a Milli-Q Ultra-pure water system (Millipore, Billerica, USA); formic acid was of HPLC grade, and obtained from Honeywell Company (Morristown, New Jersey, USA); leucine enkephalin was purchased from Sigma-Aldrich (St. Louis, MO, USA). All other reagents were HPLC grade.

### Sample preparation

The urine samples were centrifuged at 10,000 rpm for 10 min at 4°C to remove any solid debris. Fractions (50 mL) of the urine supernatants were then stored at − 80°C until UPLC-Q-TOF-HDMS analysis. Thawed urine samples were collected after centrifugation at 13,000 rpm for 10 minutes at 4°C, and the supernatant was then filtered through a syringe filter (0.22 µm), 5 µL of the supernatant were injected into the UPLC-Q-TOF-HDMS.

### Metabolic profiling

#### **Chromatographic condition**

The human urine metabolite profiling was performed with a Waters ACQUITY UPLC system (Waters Corp., Milford, USA) coupled with TOF-MS. Chromatography was carried out with an ACQUITY BEH C_18_ chromatography column (2.1 mm×100 mm, 1.7 µm). The column temperature was maintained at 45°C, and then gradient mobile phase conditions was composed of phase A (water with 0.1% formic acid) and phase B (acetonitrile containing 0.1% formic acid). A Waters Acquity UPLC BEH C_18_ (2.1 mm i.d. ×100 mm ACQUITY) column packed with 1.7 mm beads was used to separate the molecules in the biofluids set. The gradient for the urine sample was as follows: 0–5 min, 1–25% B; 5–9 min, 25–50% B; 9–9.1 min, 50–99% B; 9.1–11 min, 99% B; 11–11.1 min, 99–1% B; 11.1–13 min, 1% B. The injection volume was 5 µL and the flow rate of the LC system was 0.5 mL/min. The eluent was introduced to the mass spectrometry directly, i.e. without a split.

#### **TOF-MS condition**

MS system was operated using the ESI^+^ and ESI^−^ mode and the mass range was set at 100–1000 m/z in the full scan mode. The optimal capillary voltage was set at 3200 V, and cone voltage at 35 V. Nitrogen was used as the dry gas, the desolvation gas flow rate was set at 500 L/h, and cone gas flow was maintained at 50 L/h. The desolvation temperature was set at 350°C, and source temperature at 110°C. The scan time and inter-scan delay were set to 0.4 s and 0.1 s, respectively. Leucine enkaphalin was used as the reference compound (positive ion mode ([M+H]^+^ = 556.2771) and [M−H]^−^ = 554.2615) at a concentration of 0.2 ng/mL under a flow rate of 100 µl·min^−1^, to ensure accuracy and reproducibility during the MS analysis. The data were collected in the centroid mode, and the LockSpray frequency set at 10 s and averaged over 10 scans for correction.

#### **Multivariate data analysis**

Multivariate data was analyzed by EZinfo software (Waters Corp., Milford, USA). The unsupervised segregation was checked by principal component analysis (PCA). Potential markers of interest were extracted from the combining S- and VIP- plots from the OPLS analysis, and markers were chosen based on their contribution to the variation and correlation in the data set.

### Biomarkers identification

MassFragment™ application manager (Waters Corp., Milford, USA) was used to facilitate the MS/MS fragment ion analysis process by way of chemically intelligent peak-matching algorithms. The identities of the specific metabolites were confirmed by comparison of their mass spectra and chromatographic retention times with those obtained using commercially available reference standards.

### Construction of metabolic pathway

Construction, interaction and pathway analysis of potential biomarkers of HBV patients was performed on database source including the KEGG (http://www.genome.jp/kegg/) to identify the top altered pathways analysis and visualization.

### Statistical Analysis

SPSS 17.0 for Windows was used for the statistical analysis. The data were analysed using the Wilcoxon Mann-Whitney Test, with p<0.05 set as the level of statistical significance. MetaboAnalyst data annotation approach was used for the hierarchical clustering analysis (HCA) and significance analysis for microarrays (SAM). Receiver operating characteristic curve (ROC) was made by using the SPSS 17.0 software. The optimized cutoff values in this study were those corresponding with the highest accuracy (maximum sensitivity and specificity).

## Results

### Analysis of metabolic pattern

**Figure 1:**
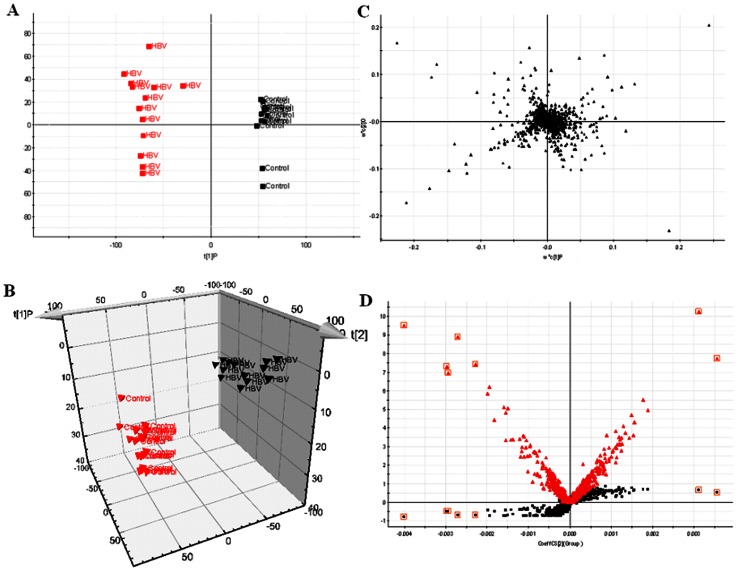
Metabolomic profiling of HBV. OPLS-DA model results for HBV group in positive mode (A). 3-D of OPLS-DA model for HBV group (B). Loading plot of OPLS-DA of HBV in positive mode (C). Panel D shows the combination of S- and VIP-score plots constructed from the supervised OPLS analysis of urine (ESI+ mode). Ions with the highest abundance and correlation in the HBV group with respect to the controls are present on the upper far right hand quadrant, whereas ions with the lowest abundance and correlation in the HBV group with respect to the control group are residing in the lower far left hand quadrant.

**Figure 2:**
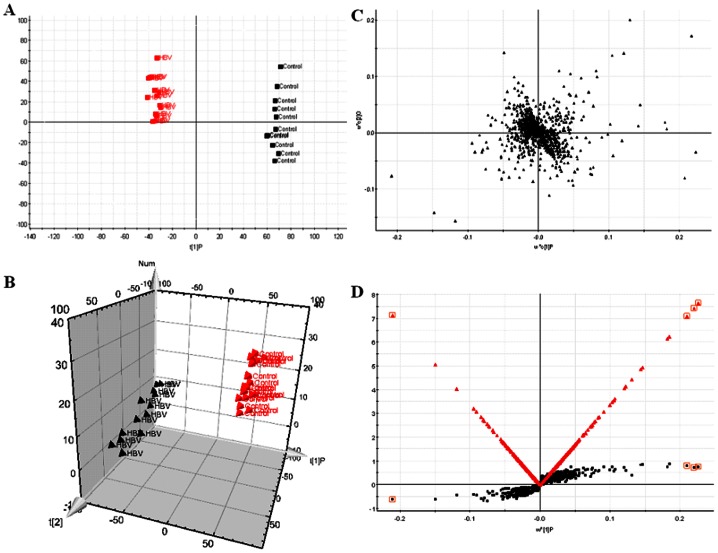
Metabolomic profiling of HBV. OPLS-DA model results for HBV group in negative mode (A). 3-D of OPLS-DA model for HBV group (B). Loading plot of OPLS-DA of HBV in positive mode (C). Panel D shows the combination of S- and VIP-score plots constructed from the supervised OPLS analysis of urine (ESI^−^ mode).

**Figure 3:**
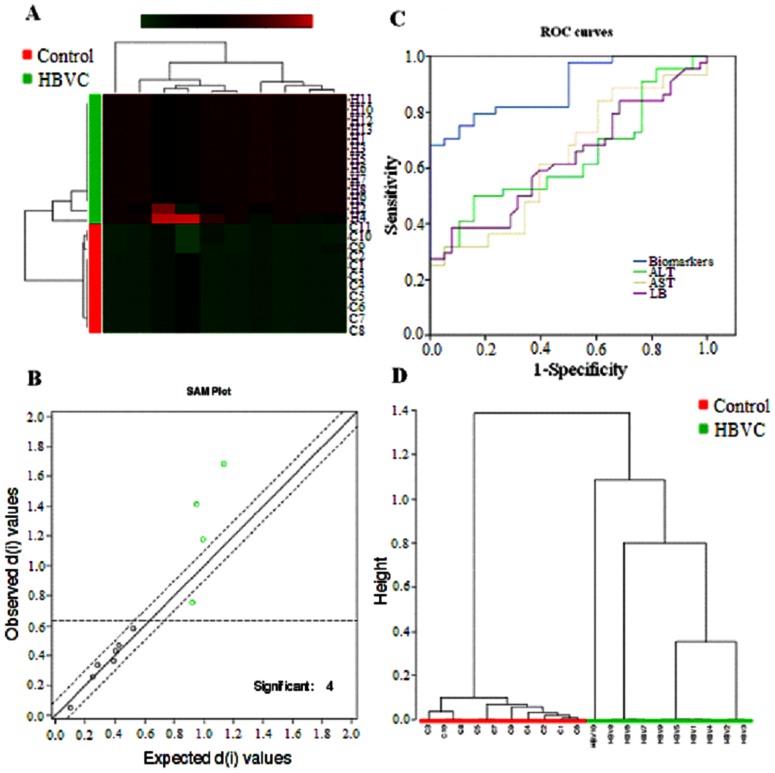
Heatmap visualization and hierarchical clustering analysis with MetaboAnalyst's data annotation tools constructed based on the differential metabolites of importance for the urine of HBV (A). Rows: samples; Columns: metabolites; Color key indicates metabolite expression value, blue: Lowest, red: highest. The significance analysis for microarrays method used to select 4 marker metabolites (B). ROC curves for the diagnosis between controls and HBV patients using data from conventional clinical chemistry markers and the metabolic markers (C). The unsupervised clustering dendrogram of prediction and diagnostic potential of the marker metabolites, tested our approach using a second set of HBV (n = 10) patients and control subjects (n = 10) to be blindly selected (D).

UPLC–MS based metabolomic approach was carried out to differentiate HBV patients from controls. OPLS-DA showed clear separation between the HBV groups and healthy group in both positive (Fig. 1A) and negative ion modes (Fig. 2 A). Trajectory analysis on 3-D score plots for the the control and HBV groups showed obvious segregation (Fig. 1B and 2 B). Overall 7319 retention time-exact mass pairs were determined in urine sample profile. From the corresponding the loading plots, the ions furthest away from the origin may be therefore regarded as the differentiating metabolites (Fig. 1C, and 2C). Combining the results of S- and VIP- plots from the OPLS analysis (Fig. 1D, and 2D), UPLC-HDMS provided the retention time, precise molecular mass and MS/MS data for the identification of biomarkers. A Wilcoxon Mann-Whitney test was performed and it was found that 11 ions (VIP>7) significantly changed (p<0.05) between the disease and the control groups. Finally, potential biomarkers of significant contribution were listed in table 1. MetaboAnalyst's data annotation tools revealed differences between the two groups (Fig. 3). Heatmap visualization, commonly used for unsupervised clustering, (Fig. 3A) for the HBV and controls showed distinct segregation.

**Table 1:** Identification of urinary biomarkers in HBV cases.

No.	Rt	[M+H]^+^	[M−H]^−^	Formula	Name	Trend	VIP	P-value
1	2.78	181.0415		C_9_H_12_N_2_O_2_	Tyrosinamide	↑	7.31	0.01
2	3.39	265.1142		C_13_H_16_N_2_O_4_	Alpha-N-Phenylacetyl-L-glutamine	↓	10.26	0.01
3	3.61	277.0288		C_10_H_16_N_2_O_5_S	Biotin sulfone	↑	7.44	0.00
4	5.34	117.0837		C_6_H_13_O_2_	Hexanoic acid	↑	9.52	0.02
5	6.19	341.1915		C_21_H_40_O_3_	5-oxo-heneicosanoic acid	↓	7.01	0.00
6	7.38	502.0894		C_18_H_35_N_3_O_13_	D-Glucosaminide	↓	7.73	0.00
7	9.35	144.0735		C_10_H_10_N	1-aminonaphthalene	↑	8.89	0.01
8	2.59		172.6996	C_6_H_6_O_4_S	Phenyl sulfate	↓	6.13	0.04
9	3.36		383.3392	C_27_H_43_O	7-Dehydrocholesterol	↑	5.06	0.00
10	3.81		186.7564	C_9_H_16_O_4_	Azelaic acid	↑	7.09	0.01
11	4.32		192.6908	C_10_H_9_NO_3_	2-Methylhippuric acid	↓	7.43	0.00

### Identification and selection of biomarkers

According to the above OPLS-DA model, a total of 11 variables (ions) with a VIP>7 were selected. Here, a biomarker with Rt-m/z of 2.78–181.0415 in positive ion mode was detailed as an example to illustrate the identification process. Using a mass tolerance of 5mDa, C_9_H_12_N_2_O_2_ was located as the candidate because of its high mass accuracy (−0.2 mDa or −0.5 ppm) and low i-fit value (1.6) among the possible chemical formulas. And then, C_9_H_12_N_2_O_2_ was input in the METLIN, (http://metlin.scripps.edu/) for possible compound, and tyrosinamide was finally emerged, which was further confirmed by comparing it to its authentic standards. At the defined operational conditions, all of the metabolites in a total ion chromatogram were extracted and aligned using the EZinfo software 2.0 (Waters Corp., Milford, USA), 11 discriminant metabolites were identified (table 1). Finally, these metabolites were selected as candidate markers for further validation. The SAM method was used to select the most discriminant and interesting biomarkers. The results indicated that biotin sulfone, 5-oxo-heneicosanoic acid, d-Glucosaminide and 2-methylhippuric acid were the most significant differential metabolites for the classification of the HBV and the control (Fig. 3B).

### Validation of metabolite markers

In order to test the usefulness of marker metabolites to discriminating HBV from controls, a second set of HBV (n = 10) patients and control subjects (n = 10) to be blindly selected. Four marker metabolites had a sensitivity of 92.83% and a specificity of 91.27% (the cutoff value: 0.66, Fig 3C), whereas the results of ALT, AST and LB (the cutoff value: 0.54) for these patients were 64.53%, 59.91% and 63.10%, respectively. Furthermore, the ROC curve analysis of the 4 marker metabolites yielded an AUC of 0.807, which was greater than that of ALT (0.619), AST (0.624) and LB (0.628), respectively. The unsupervised clustering further showed that using 4 metabolites biomarkers we can discern the controls and HBV cases being classified correctly (Fig. 3D).

### Metabolic pathway and function analysis

**Figure 4:**
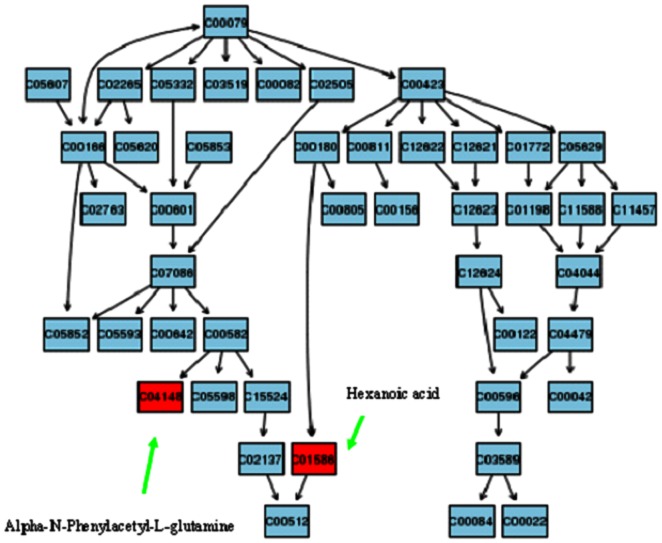
Construction of the altered phenylalanine metabolism pathways in human HBV disease. The map was generated using the reference map by KEGG. CO represents entry number of compound.

The detailed analysis of the most relevant pathways of HBV were performed by MetaboAnalyst's tool that is a free, web-based tool that combines result from powerful pathway enrichment analysis involved in the conditions under study. Consequently, potential metabolic pathway with MetaboAnalyst revealed that metabolites which were identified together are important for the host response to HBV. Three metabolic pathway of importance including metabolism of xenobiotics by cytochrome P450, phenylalanine metabolism, amino sugar and nucleotide sugar metabolism were found to be disturbed in HBV patients. The altered phenylalanine metabolism pathways with higher score was generated using the reference map by searching KEGG (Fig. 4).

## Discussion

Infections with HBV represent a significant cause of morbidity and mortality worldwide and constitute a problem of global public health importance. The WHO estimates that, currently, more than two billion individuals among the global population have been infected with HBV and of these, approximately 360 million are chronically infected [21]. Furthermore, it is estimated that as much as one third of the world population have been exposed to HBV [22]. Invasive LB is presently the best means of diagnosing cirrhosis, but it carries a significant risk and has well recognised limitations such as sampling error, hence the importance in developing early diagnosis biomarkers, hindering the comprehensive understanding to this severe disease[23]. Because of limitations in conventional approaches, several noninvasive tests have been developed for this purpose. Considering these limitations and patients' reluctance to undergo a LB, there is a need for the development of novel noninvasive techniques to detect early liver damage. With recent advances in metabolomics, biomarkers can now be identified by discovery strategies that are not limited by our existing biological knowledge. With this aim, we performed a pilot metabolomic study to assess this as a strategy for urine marker detection in patients suffering from HBV-associated liver cirrhosis.

Currently, researchers have focused on protein and gene levels, while the associated metabolic variations have been poorly understood. In our study, an UPLC–TOF/MS-based urine metabolomics approach coupled with multivariate statistical methods provide a powerful approach to clearly differentiate patients with HBV from matched control subjects and identify the potential biomarkers. Results indicate that OPLS-DA revealed an evident and statistically significant separation between the HBV and control samples. Differential metabolites identified from the metabolomic analysis suggest a disrupted the metabolism of xenobiotics by cytochrome P450, phenylalanine metabolism, amino sugar and nucleotide sugar metabolism. Interestingly, 11 distinct metabolites identified from these pathways, many are in various stages of progress at the HBV. Further study of these metabolites may facilitate the development of non-invasive biomarkers and more efficient therapeutic strategies for HBV. Computational systems analysis with MetaboAnalyst tools provides a powerful approach to metabolic profiling of urine to differentiate patients from control subjects. The patients with HBV were easily distinguished from control subjects by Heatmap approach when using autoscaling methods. Discover the early biomarkers of the occurrence and development of HBV would be helpful for the prevention and treatment. The study was firstly focused on the systemic metabolic changes in the human urine metabolome and the targeted metabolomics provides a global view to monitor the dynamic metabolic alterations of HBV processes.

SAM method was used to select the most most discriminant and interesting biomarkers. Biotin sulfone, 5-oxo-heneicosanoic acid, d-glucosaminide and 2-methylhippuric acid were the most significant differential metabolites for the classification of the HBV and the control (Fig. 3B). Biotin sulfone is a natural biotin metabolite in human urine. D-dlucosaminide is an intermediate in the human aminosugars metabolism. Methylhippuric acid is an acyl glycine that is normally minor metabolites of fatty acids. However, the excretion of certain acyl glycines is increased in several errors of metabolism. In certain cases the measurement of these metabolites in body fluids can be used to diagnose disorders associated with mitochondrial fatty acid beta-oxidation. To determine whether the 4 metabolic markers that were identified could be used for clinical HBV diagnosis, we performed a preliminary validation using the sera from 10 patients with HBV diseases. Compared with the traditional HBV biomarker ALT and AST, the combinational metabolic markers showed a better sensitivity. As shown in Fig. 3C, when biotin sulfone, 5-oxo-heneicosanoic acid, d-Glucosaminide and 2-methylhippuric acid, were used as biomarkers, the HBV-positive group could be clearly separated from the control group in HBV prediction. The unsupervised clustering further showed that using four metabolites biomarkers we can discern the controls and HBV cases being classified correctly (Fig. 3D). HBV patients was successfully differentiated from healthy controls with an accuracy of 100% using a panel of metabolite markers. Our work shows that metabolomic profiling approach is a promising screening tool for the diagnosis of HBV patients. The utility of urine metabolome diagnostics for HBV is successfully demonstrated in this study and these results suggest that metabolomics approach complements the clinical detection of HBV, leading to an improved disease diagnosis and prognosis.

Identification of metabolic biomarkers has the potential to improve diagnostic, prognostication and therapy [24], [25]. Deciphering the molecular networks that distinguish diseases may lead to the identification of critical biomarkers for diseases [26]. Metabolic pathways incorporate complex interaction networks, are usually considered to provide information on mechanisms of disease and have become a common and probably the most popular form of representing biochemical information for hypothesis generation and validation. Pathway and network analyses have both been applied to metabolomic analysis, which vastly extends its clinical relevance and effects. Thus, metabolomics holds promise for early diagnosis, increased choice of therapy and the identification of new metabolic pathways that could potentially be targeted in liver disease. Based on the KEGG, a detailed construction of the the altered tyrosine metabolism pathways map with higher score is shown in Fig. 4. Result suggest that these target pathways show the marked perturbations over the entire time-course of HBV and could contribute to the development of HBV. These biochemical changes are helpful to understand the key features of HBV. In addition, these metabolic features provided useful clues for future mechanism exploration and identification of therapeutic targets of HBV.

In the present study, a full spectrum of metabolic aberrations that are directly linked to HBV patients is critical for developing and deploying molecular diagnostic and therapeutic approaches that will significantly improve patient survival. Our studies examined a range of metabolites that represented the metabolic regulation of HBV patients and illustrated the ability of metabolomics to identify the potential biomarkers of HBV. Here, we observed a number of dysregulated metabolic pathways, such as metabolism of xenobiotics by cytochrome P450, phenylalanine metabolism, amino sugar and nucleotide sugar metabolism. A panel of metabolite markers composed of biotin sulfone, 5-oxo-heneicosanoic acid, d-glucosaminide and 2-methylhippuric acid was selected, which was able to discriminate HBV subjects from their healthy counterparts. These potential metabolite markers provide a novel and promising molecular diagnostic approach for the early detection of HBV. The results not only indicated that urine metabolomic methods had sufficient sensitivity and specificity to distinguish HBV from healthy controls, but also have the potential to be developed into a clinically useful diagnostic tool, and could also contribute to a further understanding of disease mechanisms.

## Conclusions

HBV infection is a serious global heath problem. It is crucial to monitor this disease more closely with a non-invasive marker in clinical trials. Metabolomics provides a powerful approach to discover diagnostic and therapeutic biomarkers by analyzing global changes in an individual's metabolic profile. The aim of the current study is to explore urine metabolomics as a disease diagnostic and stratification tool for HBV. A panel of 11 differential metabolites associated with HBV were selected and identified using OPLS-DA model with S-plot. More importantly, four marker metabolites that provided the effective diagnosis for human HBV. Interestingly, metabolism of xenobiotics by cytochrome P450, phenylalanine metabolism, amino sugar and nucleotide sugar metabolism was found that the most altered functional pathway associated with HBV. These results reveal that the metabolomics strategy is a powerful tool to gain insight into the mechanism of HBV at the metabolic level. In conclusion, it not only indicated that metabolomic methods had sufficient sensitivity and specificity to distinguish HBV from healthy controls, but also have the potential to be developed into a clinically useful diagnostic tool, and could also contribute to a further understanding of disease mechanisms, show the promise of this approach in developing a profile for earlier detection.

## Supporting Information

Table S1 **Clinical characteristics and liver fuction of the study population.**
(DOC)

## References

[1] Wedemeyer H, Yurdaydìn C, Dalekos GN, Erhardt A, Çakaloğlu Y, et al (2011) Peginterferon plus adefovir versus either drug alone for hepatitis delta. N Engl J Med 364: 322–331. 21268724 10.1056/NEJMoa0912696

[2] Zheng D, Zhang S, Zhou X, Wang C, Xiang P, et al (2012) The FgHOG1 Pathway Regulates Hyphal Growth, Stress Responses, and Plant Infection in Fusarium graminearum. PLoS One 7: e49495. 23166686 10.1371/journal.pone.0049495 PMC3498113

[3] Amantonico A, Urban PL, Zenobi R (2010) Analytical techniques for single-cell metabolomics: state of the art and trends. Anal Bioanal Chem 398: 2493–2504. 20544183 10.1007/s00216-010-3850-1

[4] Zhang A, Sun H, Yan G, Han Y, Ye Y, et al (2013) Urinary metabolic profiling identifies a key role for glycocholic acid in human liver cancer by ultra-performance liquid-chromatography coupled with high-definition mass spectrometry. Clin Chim Acta 418C: 86–90. 10.1016/j.cca.2012.12.024 23313056

[5] Zhang A, Sun H, Wang P, Han Y, Wang X (2012) Recent and potential developments of biofluid analyses in metabolomics. J Proteomics 75: 1079–1088. 22079244 10.1016/j.jprot.2011.10.027

[6] Zhang A, Sun H, Wang X (2012) Power of metabolomics in biomarker discovery and mining mechanisms of obesity. Obes Rev. doi: 10.1111/obr.12011. 10.1111/obr.12011 23279162

[7] Zhang A, Sun H, Wu X, Wang X (2012) Urine metabolomics. Clin Chim Acta 414: 65–69. 22971357 10.1016/j.cca.2012.08.016

[8] Zhang A, Sun H, Han Y, Yuan Y, Wang P, et al (2012) Exploratory urinary metabolic biomarkers and pathways using UPLC-Q-TOF-HDMS coupled with pattern recognition approach. Analyst 137: 4200–4208. 22852134 10.1039/c2an35780a

[9] Wang X, Zhang A, Wang P, Sun H, Wu G, et al. (2013) Metabolomics coupled with proteomics advancing drug discovery towards more agile development of targeted combination therapies. Mol Cell Proteomics. doi: 10.1074/mcp.M112.021683. 10.1074/mcp.M112.021683 PMC3650334 23362329

[10] Walsh BH, Broadhurst DI, Mandal R, Wishart DS, Boylan GB, et al (2012) The metabolomic profile of umbilical cord blood in neonatal hypoxic ischaemic encephalopathy. PLoS One 7: e50520. 23227182 10.1371/journal.pone.0050520 PMC3515614

[11] Wang X, Zhang A, Sun H (2012) Future perspectives of Chinese medical formulae: chinmedomics as an effector. OMICS 16: 414–21. 22734809 10.1089/omi.2011.0138 PMC3394855

[12] Wang X, Wang Q, Zhang A, Zhang F, Zhang H, et al (2013) Metabolomics study of intervention effects of Wen-Xin-Formula using ultra high-performance liquid chromatography/mass spectrometry coupled with pattern recognition approach. J Pharm Biomed Anal 74: 22–30. 23245229 10.1016/j.jpba.2012.10.009

[13] Hall JA, Jewell DE (2012) Feeding healthy beagles medium-chain triglycerides, fish oil, and carnitine offsets age-related changes in serum Fatty acids and carnitine metabolites. PLoS One 7: e49510. 23145181 10.1371/journal.pone.0049510 PMC3492282

[14] Zhang A, Sun H, Wang X (2012) Power of metabolomics in diagnosis and biomarker discovery of hepatocellular carcinoma. Hepatology doi: 10.1002/hep.26130. 10.1002/hep.26130 23150189

[15] Zhang A, Sun H, Wang X (2012) Saliva metabolomics opens door to biomarker discovery, disease diagnosis, and treatment. Appl Biochem Biotechnol 168: 1718–1727. 22971835 10.1007/s12010-012-9891-5

[16] Dove AD, Leisen J, Zhou M, Byrne JJ, Lim-Hing K, et al (2012) Biomarkers of whale shark health: a metabolomic approach. PLoS One 7: e49379. 23166652 10.1371/journal.pone.0049379 PMC3499553

[17] Wang X, Yang B, Zhang A, Sun H, Yan G (2012) Potential drug targets on insomnia and intervention effects of Jujuboside A through metabolic pathway analysis as revealed by UPLC/ESI-SYNAPT-HDMS coupled with pattern recognition approach. J Proteomics 75: 1411–1427. 22134358 10.1016/j.jprot.2011.11.011

[18] van Wietmarschen HA, Dai W, van der Kooij AJ, Reijmers TH, Schroën Y, et al (2012) Characterization of rheumatoid arthritis subtypes using symptom profiles, clinical chemistry and metabolomics measurements. PLoS One 7: e44331. 22984493 10.1371/journal.pone.0044331 PMC3440441

[19] Wong VW, Wong GL, Chu WC, Chim AM, Ong A, et al (2012) Hepatitis B virus infection and fatty liver in the general population. J Hepatol 56: 533–540. 22027575 10.1016/j.jhep.2011.09.013

[20] Lee SA, Kim K, Kim H, Kim BJ (2012) Nucleotide change of codon 182 in the surface gene of hepatitis B virus genotype C leading to truncated surface protein is associated with progression of liver diseases. J Hepatol 56: 63–69. 21827734 10.1016/j.jhep.2011.06.028

[21] Chen J, Wen H, Liu J, Yu C, Zhao X, et al (2012) Metabonomics study of the acute graft rejection in rat renal transplantation using reversed-phase liquid chromatography and hydrophilic interaction chromatography coupled with mass spectrometry. Mol Biosyst 8: 871–8878. 22237823 10.1039/c2mb05454j

[22] McMahon MA, Jilek BL, Brennan TP, Shen L, Zhou Y, et al (2007) The HBV drug entecavir - effects on HIV-1 replication and resistance. N Engl J Med 356: 2614–2621. 17582071 10.1056/NEJMoa067710 PMC3069686

[23] Fabris C, Falleti E, Cussigh A, Bitetto D, Fontanini E, et al (2011) IL-28B rs12979860 C/T allele distribution in patients with liver cirrhosis: role in the course of chronic viral hepatitis and the development of HCC. J Hepatol 54: 716–722. 21146242 10.1016/j.jhep.2010.07.019

[24] Zhang A, Sun H, Dou S, Sun W, Wu X, et al (2013) Metabolomics study on the hepatoprotective effect of scoparone using ultra-performance liquid chromatography/electrospray ionization quadruple time-of-flight mass spectrometry. Analyst 138: 353–361. 23152956 10.1039/c2an36382h

[25] Wang X, Zhang A, Han Y, Wang P, Sun H, et al (2012) Urine metabolomics analysis for biomarker discovery and detection of jaundice syndrome in patients with liver disease. Mol Cell Proteomics 11: 370–80. 22505723 10.1074/mcp.M111.016006 PMC3412968

[26] Meissen JK, Yuen BT, Kind T, Riggs JW, Barupal DK, et al (2012) Induced pluripotent stem cells show metabolomic differences to embryonic stem cells in polyunsaturated phosphatidylcholines and primary metabolism. PLoS One 7: e46770. 23077522 10.1371/journal.pone.0046770 PMC3471894

