# Supplementary material for: Urinary Metabolic Biomarker and Pathway Study of Hepatitis B Virus Infected Patients Based on UPLC-MS System
Source: PLoS One. 2013 May 16;8(5):e64381. doi: 10.1371/journal.pone.0064381 (PMC3655955; doi:10.1371/journal.pone.0064381)
Supplement: Table S1 — Clinical characteristics and liver fuction of the study population. (DOC) [file pone.0064381.s001.doc]

**Table S1**. Clinical characteristics and liver fuction of the study population.

| Samples | Control (n=11) | HBV(n=13) |
| --- | --- | --- |
| Sex (F/M) | 5/6 | 6/7 |
| Age | 45±3 | 44±6 |
| BMI(kg/m2) | 22.31±1.21 | 22.49±2.03 |
| HBsAg(negative/positive) | negative | positive |
| ALT (U/L) | 47.95±25.13 | 174.89±65.02 |
| AST (U/L) | 56.73±19.56 | 99.43±37.81 |
| ALP(U/L) | 74.18±26.37 | 135.76 ± 17.39 |
| ALB(g/L) | 48.38±14.22 | 30.63±5.77 |
| Total bilirubin (mg/dL) | 24.15±2.45 | 89.26±17.19 |
